# Toward understanding apoplastic freezing under negative pressure

**DOI:** 10.1111/nph.70538

**Published:** 2025-09-12

**Authors:** Stephen Ingram, Alessandro Zanetti, Linnea Mustonen, Ana A. Piedehierro, Ari Laaksonen, Anna Lintunen

**Affiliations:** ^1^ Institute for Atmospheric and Earth System Research/Physics, University of Helsinki Gustaf Hällströmin katu 2 Helsinki 00560 Finland; ^2^ Institute for Atmospheric and Earth System Research/Forestry, University of Helsinki Gustaf Hällströmin katu 2 Helsinki 00560 Finland; ^3^ Finnish Meteorological Institute Erik Palmenin aukio 1 Helsinki 00560 Finland; ^4^ Department of Technical Physics University of Eastern Finland Yliopistonrinne 3 Kuopio 70211 Finland

**Keywords:** apoplastic freezing, frost tolerance, ice nucleation, negative pressure, xylem nanobubbles

## Abstract

- The impact of negative liquid pressure on the freezing of xylem sap is a unique scientific problem. Equilibrium thermodynamics would suggest that, due to the density anomaly of ice, sap under tension should freeze at higher temperatures than under positive pressure. Yet, the opposite is observed in tree branches: freezing temperatures decrease as the pressure becomes more negative.
- Using a cold stage array, we measured the freezing points of sap samples extracted from *Pinus sylvestris* branches dehydrated to varying negative pressures and compared them with the freezing points of the sap‐filled sample branches with similar negative pressures.
- We find that the freezing onset of extracted sap is *c.* 10 degrees lower than inside the branches, and uncorrelated with the water potential that was present before extraction. Taken together, these results strongly suggest that supercooling under tension is a purely physical phenomenon and that nucleation is initiated on the surface of the xylem tissue.
- By drawing an analogy with aerosol science, we propose that pores in the conduit walls may form either an ice embryo or a gas nanobubble, with the competition between the two determining the macroscopic freezing point. A phenomenological model based on the above mechanism reproduces the observed branch freezing onset temperatures within experimental uncertainty.

## Introduction

Vascular plants transport water against gravity in a metastable state, variously called tension, water potential, and negative liquid pressure. Upward sap flow through plant stems has been studied for over a century, and is now collectively described by the cohesion–tension theory (Shi *et al*., 2020, Pockman *et al*., 1995).

Water under tension has been shown to exhibit several anomalous properties, such as its kinetic and thermodynamic quantities inverting. For example, the Tolman length (Tolman, 1949), a measure of the curvature dependence of surface tension, has been found to be negative under negative pressure (Azouzi *et al*., 2013), suggesting that concave surfaces (i.e. bubbles) are stabilized in this regime, as opposed to convex droplets. The speed of sound in liquid water normally increases with temperature, but when the water is stretched, it first decreases to a minimum before increasing (Pallares *et al*., 2016). Conversely, both density and compressibility approach maxima under tension (Holten *et al*., 2017). Due to the scarcity of experimental data, the shapes of these curves (and the so‐called line of density maxima) have been the subject of some debate (Stanley *et al*., 2002; Lu *et al*., 2016). In fact, the equation of state of water at extreme negative pressures is still unclear (Pallares *et al*., 2014), including a possible liquid–liquid critical point and/or spinodal decomposition occurring before cavitation.

It is therefore not surprising that, although the cohesion–tension theory is widely accepted within the plant physiology community, it remains controversial within the physics community (Boatwright *et al*., 2015; Vera *et al*., 2016), and is incomplete in the sense that it cannot predict the interaction of plant sap with different phases of matter. First, in artificial systems, hydraulic stability cannot be maintained under negative pressure when water is saturated with dissolved gases (Caupin & Herbert, 2006). It will boil at pressures much less negative than those accessed in vascular plants (Roper *et al*., 2012). The porous nature of xylem tissue, combined with the presence of lipid‐coated nanobubbles (Ingram *et al*., 2023), may mitigate against instability, but this is still speculative and outside the remit of the original cohesion–tension framework.

Second, the specific effects of upward sap flow on the formation of solid ice within apoplastic tree tissue remain largely unexplored. Due to the stochastic nature of heterogeneous ice formation (see, e.g. Murray *et al*., 2011; Knopf *et al*., 2020), it is impossible to predict when or where freezing will occur in the xylem network with any certainty. Some tree tissue can stay unfrozen even in deep cold, as nucleation is only influenced probabilistically by the conditions within the sap. For example, one would expect the freezing point of apoplastic water to be coupled to its chemical makeup (i.e. osmotic forces) and, as we shall soon discuss, pressure.

It is extremely important for the field of plant frost tolerance and overwintering that these phenomena are quantified more accurately. In cold environments, water in the xylem and other extracellular spaces of trees often freezes during winter. However, the water inside living cells (the symplast) must remain unfrozen, as freezing would be fatal. A process called extracellular freezing (Neuner & Pramsohler, 2006) helps protect living cells by drawing water out into the apoplast, increasing the concentration of solutes in the symplast, and lowering its freezing point. Despite this protective effect, apoplastic freezing can also be harmful. When water freezes, dissolved gases form bubbles (e.g. Lintunen *et al*., 2022), which may expand during thawing and disrupt water transport in the xylem by causing embolisms (e.g. Charra‐Vaskou *et al*., 2023). Additionally, ice crystal formation or excessive shrinkage of living cells due to water loss can lead to mechanical damage.

**Fig. 1:**
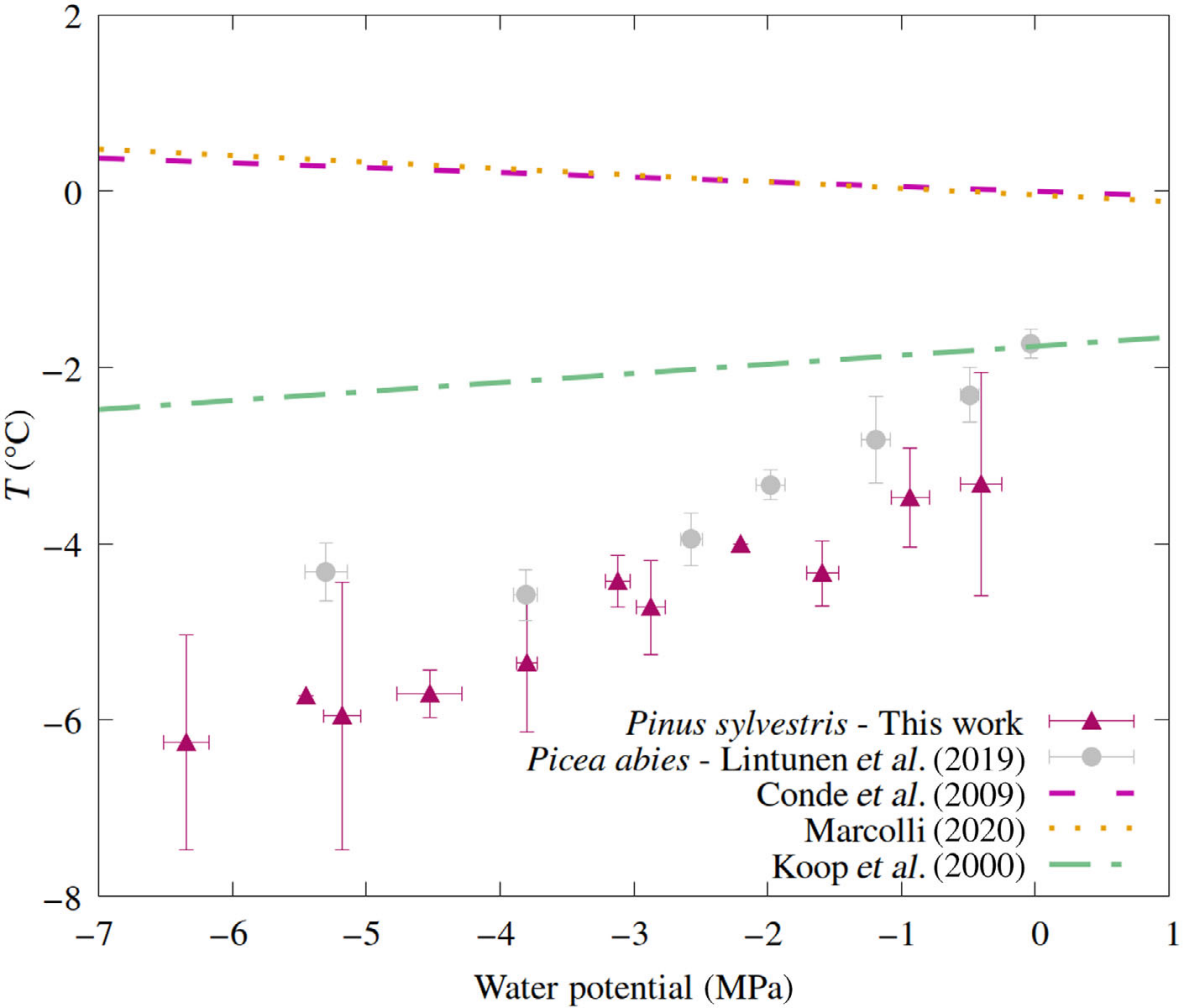
Comparison of freezing points as a function of water potential/negative pressure. Red points are new *P. sylvestris* data presented in this work; gray points are *Picea abies* from Lintunen *et al*. (2019). Red data points were averaged in bins of width 0.6 MPa, and error bars represent one SD. The Conde *et al*. (2009) and Marcolli (2020) lines (pink and orange dashed) represent the equilibrium phase boundary between liquid water and hexagonal ice. Koop *et al*. (2000) (green dashed line) is the parameterization of the effect of water activity on freezing, scaled to go through the first gray point (see Supporting Information Notes S1).

Before freezing, sap exists in a doubly metastable state where it is both supercooled below its melting temperature and stretched by the negative pressure. In other words, both of the phase transitions mentioned previously (embolism/bubble formation and freezing) are thermodynamically favored to take place in the apoplastic water. Yet they do not, or do not immediately, and the water stays liquid due to kinetic stabilization. Crucially, when it does freeze, sap exhibits heterogeneous freezing points that are proportional to the tension it experiences (Fig. 1, red and gray points), in contrast to the equilibrium phase boundary between the solid and liquid phases, which trends in the opposite direction (Fig. 1, pink and orange lines). New *Pinus sylvestris* branch freezing points are provided in unaveraged form in Supporting Information Table S1, and averaged form in Table S2.

Unlike most materials, solid water is less dense than the liquid (a phenomenon commonly referred to as the ‘density anomaly of ice’). As a result, ice can be melted by compression at below 0°C (Koop *et al*., 2000). Conversely, any force that acts to *reduce* the density of water (in this case, tension) should shift the equilibrium toward the solid, elevating the melting point (Roedder, 1967; Henderson & Speedy, 1987; Loerting *et al*., 2020).

The inverted dependence of freezing on pressure in apoplastic water has been documented in *Picea abies* (Lintunen *et al*., 2019) and *Pistacia integerrima* (Sperling *et al*., 2017) trees, as well as smaller vascular plants, such as olives (Arias *et al*., 2017) and *Larrea tridentata* (Medeiros & Pockman, 2011). Fig. 1 compares our branch freezing data thus far with varying tensions to the parameterization of Marcolli (2020), as well as theoretical predictions by Conde *et al*. (2009) using the TIP4P/2005 water model (Abascal & Vega, 2005), both predicting freezing point elevation at *P* < 0. Not only do the branches exhibit an inverted dependence of freezing on pressure in this region, but the points also do not intersect with the equilibrium phase boundary at near‐zero pressure (Fig. 1, red points).

Of course, plants are not equilibrium systems; they consume solar and chemical energy from their environment. However, sap flow is generally considered a passive process (Meinzer *et al*., 2001), and the xylem conduit walls are, to a degree, biologically inert. Dead plants have even been shown to transport water upward, in Strasberger's famous oak tree experiment (Strasberger, 1892). Basic physics would therefore predict that sap freezing should be primarily determined by the density anomaly, following a Marcolli‐like dependence on pressure, even if the freezing onset were shifted into a supercooled state.

Also included in Fig. 1 is a parameterization from Koop *et al*. (2000) of freezing based on water activity, *a*_*w*_. Activity can be used instead of the applied external pressure, as it is possible to equate the relative humidity dependence of water potential (from the cohesion‐tension theory) to the freezing point dependence of the water activity (see Notes S1). We note that, when this is done, the gradient of the line trends in the ‘correct’ direction, although it is still not sufficient to capture the observed degree of supercooling under negative pressure.

We believe that an interdisciplinary approach which combines physics and tree physiology is required to tackle this problem. To this end, we extracted sap samples from the apoplast of the boreal tree *P. sylvestris* at a range of water potentials from zero to hydraulic failure and frozen them *ex vivo* in a cold‐stage array. The aim is to test whether sap freezing is a purely physical phenomenon, initiated by active sites on the surface of the xylem conduits, or is a (bio)chemical one (determined by osmotic forces or ice nucleating particles suspended in the liquid) indirectly caused by tension.

Next, by drawing an analogy with the growth of ice on mineral particles in the atmosphere during cloud formation, we have developed a phenomenological model of xylem sap freezing under negative pressure. It assumes that pores in the conduit walls (or interconduit pits) may either form an ice embryo or a seeded nanobubble, with the competition between the two determining the macroscopic freezing point. The model relies on classical nucleation theory to determine the former and the Young–Laplace equation to determine the latter. It only requires an assumption of the mean and variance of pore sizes embedded within the conduit surface to effectively fit the model to the observed data.

We note that, in the case of *Picea abies*, the freezing points level off and become independent of water potential around the point where the branch xylem tissue begins to embolize. To more accurately capture this behavior, we introduce a variant of the model where the degree of metastability is allowed to change per MPa of pressure.

The fundamental question is whether the reversed dependence of sap freezing on pressure emerges from underlying physical processes or is a consequence of biological changes the plant undergoes that are indirectly caused by water potential. We believe that the purity of sap and the passive nature of sap ascent point toward a physical explanation. In this work, we show that it is possible to quantitatively predict freezing temperatures of tree tissue based on the characteristics of the plant hydraulic system under consideration, and indeed vice versa: to use sap freezing points to infer the structure of the xylem conduit surface. We will show that the competition between bubble formation and freezing is crucial to determining the phase behavior of apoplastic water under negative pressure.

## Materials and Methods

### *Pinus sylvestris* branch freezing

To study how branch water potential affects their freezing temperature, *Pinus sylvestris* (L.) branches were collected in January 2023 from Viikki Arboretum, Helsinki, Finland (60.219978; 25.006782; 10 m asl). Two *c*. 1‐m‐long branches were collected from each of 10 tree individuals. All samples were collected at midday, and the cut branches were brought to the laboratory.

In the laboratory, the sample branches were recut under water and kept overnight at room temperature, kept upright in a bucket full of water, and covered with black plastic bags to saturate them. After saturation, the cut ends of the branches were sealed with tape, and the branches were bench‐dehydrated under room conditions to varying water potentials. The water potential of the branches was measured daily (after keeping the branches in full darkness) from 4 to 6 needles per branch with a pressure chamber (I505D‐EXP, PMS Instrument Co., Albany, OR, USA).

During the experiment, pairs of branches with similar water potentials were stored in sealed plastic bags at +5°C to avoid further dehydration. From these pairs, one branch (randomly selected) was used for a branch freezing experiment in a test chamber and the other for sap collection.

In the branch freezing experiment, the branches were placed standing into a test chamber (Weiss Umwelttechnik WK11–340/40) in two consecutive sets (5 + 5 branches) on the same day. T‐type thermocouples were attached in contact with the bark surface to detect the heat released in freezing exotherms during the freezing of the apoplast (as described in Lintunen *et al*., 2019). The base diameter of the branches varied from 8 to 12 mm, but the thermocouples were attached to all branches at the location where the branch diameter was *c*. 6 mm. In the first step of the freezing experiment, the temperature was decreased rapidly from room temperature to 5°C. Then, the temperature was decreased to −10°C at a controlled rate of 5°C h^−1^ and held at the target temperature for 2 h.

During sap collection, the branches were re‐cut underwater from both ends to a length of 15 cm (without side branches). The bark was peeled off from the cut ends of 1 cm length, and plumbing tape was wrapped around the peeled parts to prevent any possible water connection of the bark. Then, each branch segment was attached to a tube filled with Milli‐Q water and safranin dye (to make sure we do not collect water we are feeding to the branch) and connected to a water reservoir that was lifted to a height of 1.0 m. Sap exuding from the cut branch tip was collected in sterile 1.5 ml microtubes (TreffLab CleanRoom Pure® Microtubes, Degersheim, Switzerland), after discarding the first 1–2 drops to avoid possible contamination from cutting. Ten microtubes with sap samples, each containing 1 ml of sap, were transported for further experiments. All procedure steps were done with gloves on to avoid contaminating the samples.

To gain more data on the branch freezing at even lower water potentials, another set of 20 *c*. 1‐m‐long branches were collected in February 2024 from 10 pine trees growing in the same arboretum. These branches were treated as the previously described set, that is, saturated, cut ends sealed, and bench‐dehydrated while measuring water potentials. This time, subsets of four sample branches were randomly selected at each time after reaching different target water potentials, ranging from near 0 to −7 MPa. The sets of four branches were then frozen in the test chamber with thermocouples measuring the exotherms. This time, the temperature was decreased rapidly from room temperature to 3°C, then decreased to −10°C at a controlled rate of 4°C h^−1^ and held at the target temperature for 2 h. Finally, the temperature was also increased back to 3°C at a controlled rate of 4°C h^−1^. After measuring the freezing point of the dehydrated sample branches, the branches were rehydrated and, after reaching a target water potential, refrozen to measure the freezing point again. Since the freezing point was measured twice from the same branches, a lower temperature change rate than for the first set of branches was used to avoid any possible tissue alterations during the thawing process.

The ice nucleation temperature was determined using rstudio (v.4.3.2; RStudio Team, 2025) as the temperature recorded 5 s before a detectable freezing exotherm occurred.

### Cold stage measurements

Sap samples were extracted from cut branches and then immediately transported from Viikki to the Finnish Meteorological Institute for analysis. The freezing activity of the sap samples was analyzed using an Mk‐1 droplet‐freezing cold stage (Sikora Scientific Instrumentation, Bremen, Germany). The droplet freezing experiments are performed by depositing a set of 49 sap droplets (1 μl) onto a hydrophobic glass slide placed on top of the temperature‐controlled cold plate. A high‐resolution camera captures images of the droplets to identify freezing events while the temperature is lowered and recorded. The uncertainties in the measured temperatures were found to be < 0.3°C. A 1 l min^−1^ nitrogen flow is flushed over the droplets to minimize the formation of frost. Ten‐fold dilutions with Milli‐Q water were prepared for selected samples. A minimum of 100 droplets were analyzed for each sample and dilution. During the freezing experiments, the cold stage temperature was lowered from 0°C to −30°C at a 1°C min^−1^ rate. The frozen fraction as a function of the temperature *f*_ice_ (*T*) was directly derived from the analysis of the images. The active site density per volume of sap was calculated as in Vali (1971). Although the cooling rate used here was much higher than the one used in the thermocouple experiments described in *Pinus sylvestris* branch freezing in the Materials and Methods section, we do not expect it to have significantly affected our results. According to Shardt *et al*. (2022), 1°C min^−1^ has been found to lower the measured freezing points by up to 1 degree, compared to 0.1°C min^−1^. As we will see, this is much smaller than the difference between sap and branch freezing that we observe.

## Results

One possible explanation for supercooling increasing with tension in apoplastic water is that it is a (bio)chemical phenomenon that is only indirectly caused by the negative pressure. For example, an upregulation of antifreeze proteins or flavonoids, or an increase in the electrolyte concentration. Were this the case, the effect would be somewhat irreversible; that is, the sap freezing would still show a dependence on the water potential it was held in, even in a positive pressure environment.

**Fig. 2:**
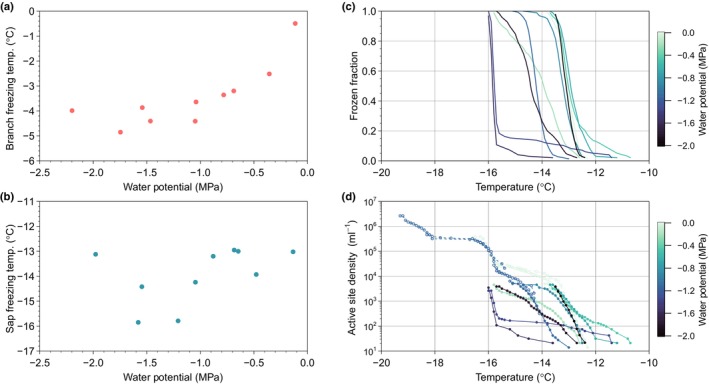
New *Pinus sylvestris* freezing data presented in this work. Left column: comparison of (a) branch and (b) sap freezing temperatures as a function of water potential of branch pairs at sap collection. Branch pairs were selected that had water potentials as close to each other as possible. Note that sap freezing temperatures are reported as the temperature at which 50% of the droplets froze. Data in panel (a) represent a subset of the red points presented in Fig. 1. Right column: cold stage freezing measurements color‐coded by the water potential of each branch at sap collection. (c) Frozen fractions. (d) Active site density normalized to sap volume. Dilution results for two samples are included as open symbols.

Our results show that this is not the case, and extracted sap freezing points are independent of the water potential that was present within the branches at the time of sap collection: the effect is not permanent (Fig. 2a,b). The sap must remain under tension and in contact with the xylem surface to exhibit any pressure dependence. Sap and branch freezing points are also directly plotted against one another in Fig. S1, and the full dataset from both experiments is provided in Table S3. The Pearson coefficient (*R* ^2^) of the comparison is 0.2279, suggesting low correlation. The second important result is that the amount of supercooling that the sap can withstand is, on average, 10 degrees more than that of the branches.

The osmotic pressure of dissolved salt ions reduces water activity (and therefore freezing points), and can even be expressed as a negative pressure. To test the magnitude of the osmotic contribution to the water potential and freezing points, the sap samples were concentrated by a factor of 10, and the cold‐stage experiment was repeated. This was found to only marginally increase the freezing points by < 0.5°C. Therefore, at least in this case, osmotic pressure does not account for a large contribution to the water potential, and we can conclude that the sap exhibits low enough osmotic pressure that it cannot account for the observed freezing behavior (Boutilier *et al*., 2014).

Conversely, diluting one of the sap samples by a factor of 1000 reduced the freezing points by *c*. 5°C, from −14°C to −19°C. This corresponds to an increase in active site density ml^−1^ of sap from 10^2^ to 10^6^ ml^−1^ (Fig. 2d). These numbers are approximately a factor of 100 smaller than the number of particles measured in sap extracted from cut branches (Guan *et al*., 2022), and would imply a concentration of nucleators on the order of picomolar (1.7 × 10^−12^ M).

### Pore freezing parametrization

Taken together, this dataset strongly suggests that the lignified walls of the xylem network are likely to be the initiator of plant apoplastic freezing during winter. Indeed, surface science would dictate that active sites for freezing are usually geometric defects (Kiselev *et al*., 2017) or specific chemical groups that are capable of bonding to water strongly during the early stages of ice formation. For example, deposition freezing on mineral particles in the atmosphere proceeds first within pores or crevices on the particle surface, as both liquid water and the ice critical embryo are stabilized by a wedge geometry (Roudsari *et al*., 2022).

**Fig. 3:**
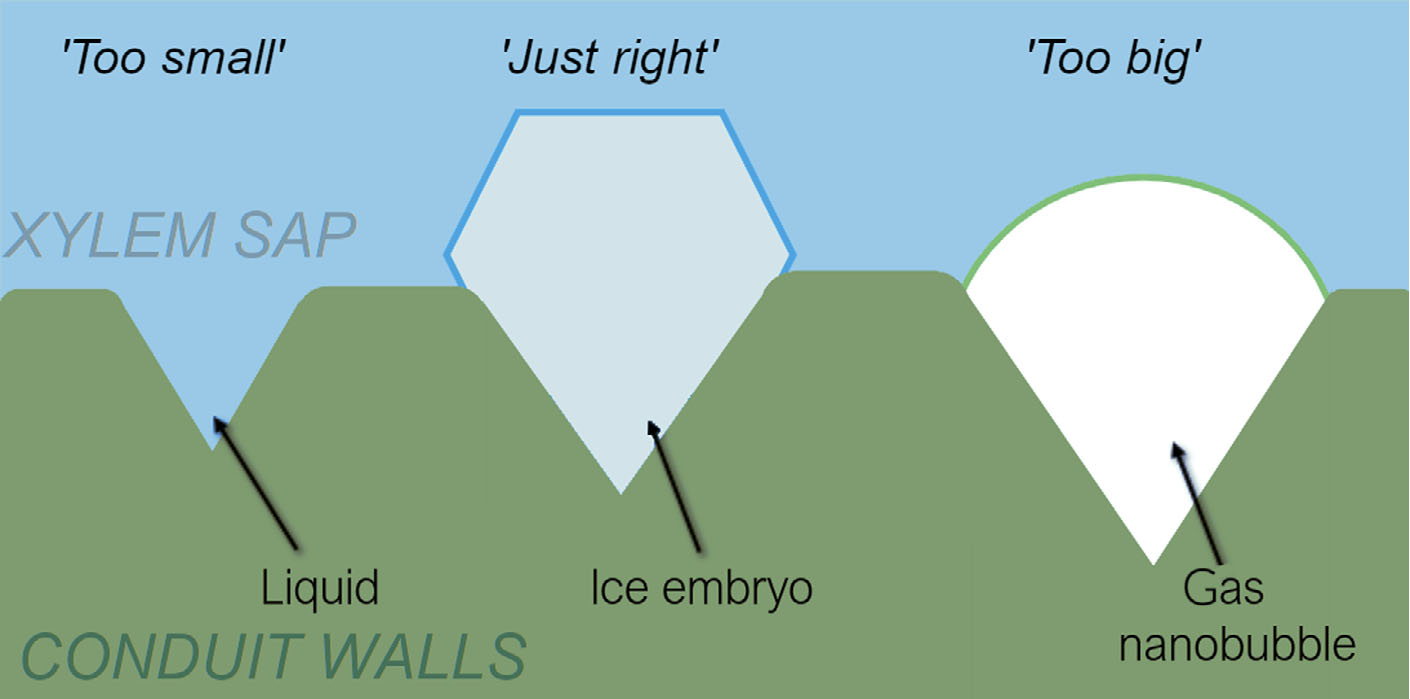
Schematic representation of the proposed ‘Goldilocks’ model underpinning the pore freezing parameterization.

In addition, the inverted dependence of branch freezing points on water potential means that ice nucleation activity is in some way inhibited by the presence of negative pressure. We therefore propose a ‘Goldilocks’ model of the freezing initiation (Fig. 3), which relies on the following assumptions:

1. There is a large population of nanoscale wedge‐like pores embedded within the xylem conduit walls. Their size distribution is lognormal.
2. Ice nucleation always begins within a pore, rapidly freezing the local conduit, and then spreading throughout the conduit network in the xylem.
3. Some proportion of the pores are ‘too big’ to freeze: their implied radius is larger than the Laplace radius of a nanobubble that would be stable at that water potential. Therefore, they are likely to be emptied of liquid water by the surrounding negative pressure.
4. Some proportion of the pores are ‘too small’: their implied radius is smaller than a critical ice embryo at that temperature. They are, therefore, functionally part of the wall and are unlikely to take part in the freezing process.
5. The remaining pores are ‘just right’: They are in a metastable region of the size distribution and so have some non‐zero probability of initiating freezing.

See Fig. S2 for a graphical representation of how this is calculated across the size distribution per pressure step. Note that assumption 3 depends on pressure, whereas assumption 4 depends on temperature. To be more specific, the Laplace radius,

(Eqn 1) $$r_{\text{Laplace}}=\frac{2\gamma_{\mathrm{wg}}}{\Delta p_{\text{Laplace}}}$$

is only weakly dependent on temperature through the water‐gas surface tension *γ*_*wg*_. Here, Δ*p*_Laplace_ is the Laplace pressure differential across the bubble's surface. By contrast, the critical embryo radius,

(Eqn 2) $$r_{\text{nucleus}}=\frac{2\gamma_{\mathrm{iw}}\nu_{i}}{-\Delta \mu_{\mathrm{iw}}}$$

is only weakly dependent on external pressure, through the molecular volume $\nu_{i}$ and ice‐water surface tension *γ*_*iw*_. Δ*μ*_*iw*_ is the chemical potential difference between the ice and water phases, which is equal to $k_{B}T\ln\frac{p_{\mathrm{wat}}\left(T\right)}{p_{\mathrm{ice}}\left(T\right)}$. *k*_*B*_ is Boltzmann's constant, and *p*_wat_ (*T*) and *p*_ice_ (*T*) are the temperature‐dependent partial vapor pressures above water and ice, respectively (here calculated using eqns 18 and 19 of Marcolli, 2020). These equations define the limits of the metastable region, and therefore the path of the system through the two axes of the phase diagram.

To initialize the model, it is also necessary to assume that the pore size most likely to freeze is the modal one. In other words, the lognormal distribution is centred at the value of Eqn 2 when *T* = *T*_onset_. Once initialized, the metastable area under the curve is then, by definition, 50%.

The total number of pores in the metastable region should then be coupled to the nucleation rate in some way. The basic assumption is that the area under the curve between the two limits should be approximately the same in each branch, at least at the temperatures at which freezing is observed. It is also possible to decouple the mean pore radius from the ice critical radius at the first pressure point, allowing a fixed, but tunable, metastable area. Note that area here refers to the geometric area between the two radii in the size distribution, rather than the physical area of the conduit surface. The effect of changing the area is shown in Fig. S3a, from 10% to 60%, as well as the effect of the variance in pore size in the fixed mode in Fig. S3b.

In situations where the nucleation rate is thought to change as a function of pressure, it is possible to run the model in a different mode: in this case, we dynamically change the width of the metastable area as a function of the negative pressure. In terms of the internal functioning of the model, it means that *r*_nucleus_ decreases at either an accelerated or decelerated rate per MPa of pressure, relative to *r*_Laplace_.

An expanded metastable region means a faster decrease in *r*_nucleus_, necessitating deeper levels of supercooling in order to initiate freezing. Conversely, a slower decrease in *r*_nucleus_ means a slower decrease in *T* as a function of pressure. We discuss possible connections between this and hydraulic vulnerability in Discussion section.

We would like to caution that, because the model is fitted to branches with measured water potentials, it inherently assumes static water potential in time and space. This is an oversimplification: xylem pressure typically shows diurnal dynamics, individual conduits experience varying pressure, and water potential becomes less negative, on average, toward the roots. We have also considered the osmotic component of water potential to be negligible, which may not always be the case.

**Fig. 4:**
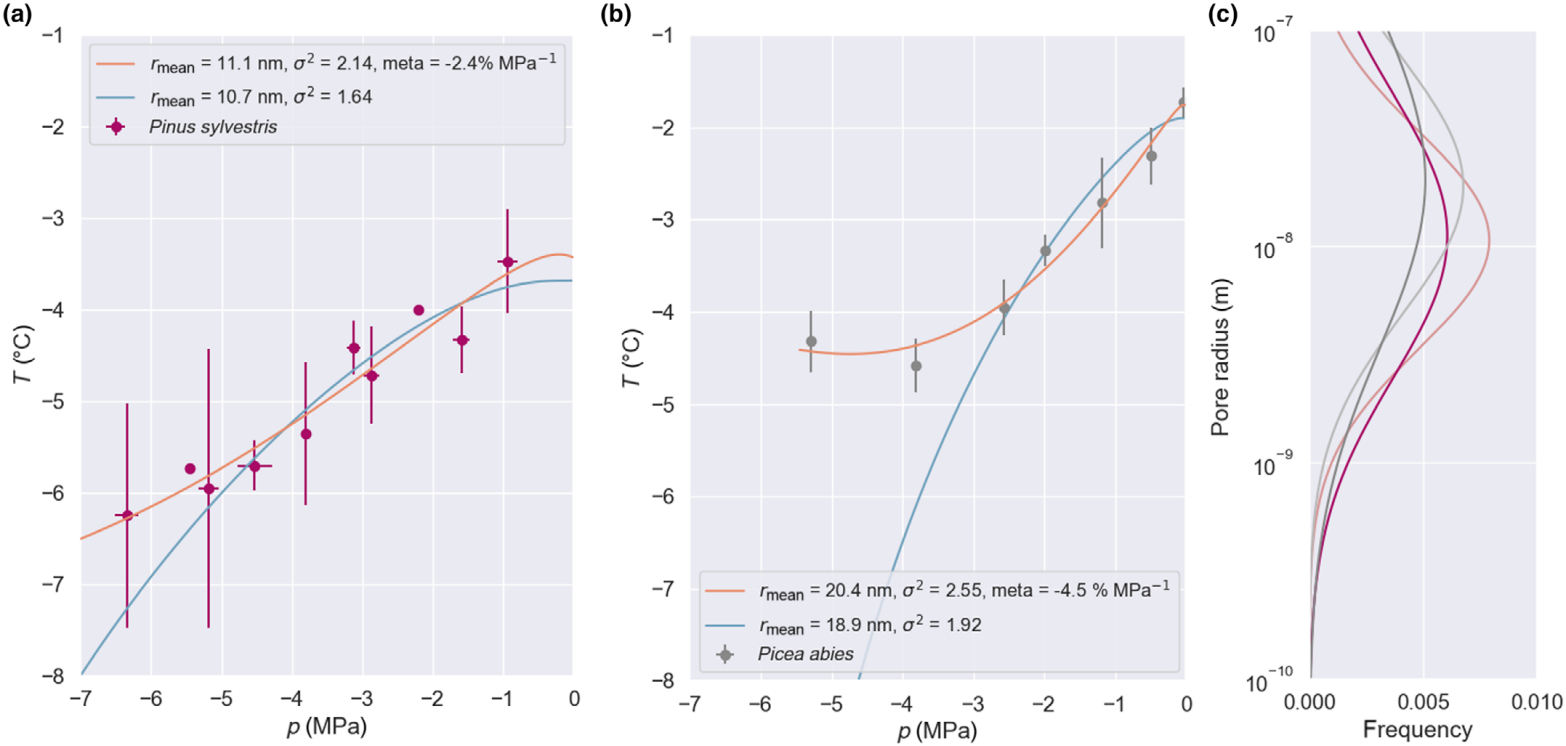
Best fits of the pore freezing model to the averaged branch freezing data for (a) *Pinus sylvestris* and (b) *Picea abies*. Blue lines are the model in fixed metastability mode, and orange lines are in variable. Error bars represent one SD of the averaged data. (c) Implied pore size distributions from the model fits for *Pinus sylvestris* (red) and *Picea abies* (gray). Faded lines represent the underlying distributions for the fixed metastability mode that produce the blue lines in (a, b).

Best fits of the pore freezing model to the branch freezing data have been produced both for *P. sylvestris* data collected in this work (Fig. 4a), and *P. abies* from Lintunen *et al*. (2019) (Fig. 4b). The onset temperature was allowed to vary by ± 0.5 K from its measured value, the variance *σ* ^2^ between 0.5 and 3.0 logarithmic units, and the metastability between −5 and +4% MPa^−1^. We find that when running the model in the fixed metastability mode (blue lines), it initially shows good agreement with experiment, given the spread of the data, before curving downward at low water potential/highly negative pressure, where the data levels off. By contrast, the variable metastability curves closely follow the experimental data for both species (orange lines). Also shown are the underlying pore size distributions, plotted on a log scale (Fig. 4c). By comparing the two species, it can be seen that an approximate factor of two increase in the mean pore size increases the onset freezing temperature by 1.5 degrees.

## Discussion

Several candidates have been proposed as the initiators of freezing in different plant types, including extrinsic (e.g. *Pseudomonas* bacteria; Cochet & Widehem, 2000) and intrinsic (e.g. in flower buds; Ishikawa *et al*., 2016) nucleators. See also the viewpoint of Lamacque *et al.* (under review) in this issue for a more detailed overview. This work does not seek to suggest that xylem pores are the sole nucleators of ice during overwintering. Rather, we wish to draw attention to the fact that the reduction in freezing points with tension is an example of ice formation being inhibited (as can be seen in Fig. 1, a lowering of the water potential by 2 MPa yielded a decrease in freezing temperature of *c*. 3°C). As such, it requires an ‘antinucleator’ to explain – something that decreases the nucleation rate and allows deeper levels of supercooling to be accessed as the pressure becomes more negative.

The presence of nanobubbles in xylem sap has been somewhat controversial, though evidence of their existence has been mounting for some time: they have been observed in frozen sap samples using electron microscopy (Schenk *et al*., 2017), their size and dynamics measured by nanoparticle tracking (Guan *et al*., 2022), and their stability under negative pressure explained by theoretical calculation (Manning, 2020; Vehmas & Makkonen, 2021; Ingram *et al*., 2024). Briefly, nanobubbles (i.e. bubbles below 1 μm in size) are produced whenever an embolized conduit is adjacent to a water‐filled one. The suction force of the negative pressure draws air through the pit membrane pores by extrusion (Schenk *et al*., 2015; Ingram *et al*., 2023). They are coated in lipids during the process, which stabilizes them with respect to expansion (due to dynamic surface tension; Ingram *et al*., 2021) and coagulation (due to their negative surface charge, causing them to repel one another; Ahmed *et al*., 2018). Once entrained, individual bubbles cannot leave the conduit they are in, but it is believed that gas is able to travel through pits, forming new bubbles in the process (Krieger *et al.*, 2022). As such, the concentration of nanobubbles is largest near embolized or cut regions of xylem, decaying with increased distance (Guan *et al*., 2022).

We wish to emphasize that the bubbles under consideration in this model are not the result of air seeding, as they usually would be through pit membranes. Instead, they are nucleated (or ‘degassed’) within the wedge geometry, for much the same reason as the ice would be: the surrounding sap is metastable with respect to ‘detaching from the walls’ (Barbosa *et al*., 2025), and the wedge geometry catalyzes the nucleation. The resultant nanobubbles would therefore not be stable floating freely, and would stay within the pore. The absence of liquid water within the pore would then render it inactive with respect to ice formation.

Relatedly, we have assumed that the nucleated nanobubbles would not be lipid‐coated and that their interfacial tension *γ*_*wg*_ would simply be that of water. Therefore, only two (or three) parameters are required to fit to experimental data: the mean pore radius (which defines *T*_onset_), the variance/SD of the pore size distribution (which defines the slope of the line through *p*, *T* space), and optionally the percentage change in metastable area MPa^−1^.

The Classical Nucleation Theory embryo radius decays rapidly with temperature, as shown in Table 1, approaching an asymptote of *c*. 3 nm. The implied mean pore radius scales with the freezing temperature at close to zero water potential in the same way. The fit values of the mean pore radii for the two species investigated herein are shown in the figure key, and are 20.4 nm for *Pinus sylvestris* and 11.1 nm for *Picea abies*. While the nanoscale structure of xylem conduits remains unmeasured, these values are consistent with the pore size found in pit membranes by Li *et al*. (2020) (5–20 nm). Since the cellulose fibers comprising pit membranes (Nishiyama *et al*., 2008) are similar in size and shape to lignin fibers comprising xylem walls (they can even interweave, for example, in lignocellulosic material), this is promising and suggests the conduit wall pore size may be similar to the pit membrane value.

**Table 1:** Classical nucleation theory critical nucleus radii, *r*_nucleus_, calculated from Eqn 2 as a function of temperature (T).

*T*(°C)	*r*_nucleus_ (nm)
0	243.4
−1	33.6
−2	18.1
−3	12.4
−4	9.4
−5	7.6
−6	6.4

Additionally, we note that the mean pore radii scale approximately with the measured branch conduit radii of the two species (Held *et al*., under review). It may therefore be the case that trees with wider vessels will freeze at higher temperatures than those with narrower ones (Lintunen *et al*., 2013), although much more data would need to be collected in order to say definitively. Interestingly, this is the opposite of what one would expect when considering simply the surface area in contact with the liquid. Normally, a larger surface area to volume ratio would promote a phase transition. Instead, here smaller conduits may contain smaller pores, which would require more supercooled sap in order for the nucleation to be initiated.

Another issue preventing further interpretation of our model is that, at present, the molecular structure of the lignified conduit coatings and pit membranes remains largely unexplored. For example, the cellulose nanofibres that comprise pits can act as either hydrophobic or hydrophilic depending on which face of the fiber is under consideration (Nishiyama *et al*., 2008), as a result of their three‐dimensional chemical structure. The nanoscale details of their interaction with water, and hence ice nucleation ability, are *a priori* unknown. We hope that, in the coming years, microscopy and molecular simulation studies will shed some light on these gaps.

There is also conflicting evidence in the literature as to whether ice nucleation in plants is the result of a single event or many (Charrier *et al*., 2017). In the fixed metastability mode, our model assumes a single event that spreads rapidly throughout the xylem network. Fitting the model to the *P. sylvestris* data with a fixed level of metastability shows a deviation at *c*. −3 MPa, where the measured freezing points level off and become independent of pressure (Fig. 4, blue lines). We observe that this decoupling of model and experiment happens around the point at which hydraulic failure occurs in both species (González‐Muñoz *et al*., 2018). We propose the following explanation.

Once the water column has lost cohesion, the xylem network splits into many independent groups of water‐filled conduits, surrounded by otherwise embolized wood. The negative pressure in the remaining liquid is likely to remain near the value it was when the hydraulic failure happened. The measured leaf water potential will continue to fall, but it is disconnected from the majority of the sap in the network.

In this scenario, many independent (microscopic) ice formation events would need to occur in order for a thermocouple to observe a macroscopic release of latent heat. Our model, operating in a fixed metastability mode, is therefore inappropriate for such a system, and it is unsurprising that the freezing points level off at that pressure.

Put another way, we suggest that post‐hydraulic failure freezing points are the result of multiple independent nucleation events. One can assume that there are as many nucleation events as there are groups of isolated conduits in the xylem network – in which case the nucleation rate is likely to be several orders of magnitude higher than when the water column was intact. As such, they should be treated by the model in the variable metastability mode.

### Conclusion

To our knowledge, this is the first attempt to theoretically explain or model freezing under tension within woody plants. By extracting sap samples from *P. sylvestris* individuals at a range of tensions, we find that the influence of water potential on freezing points in the branches was not transferred to the extracted sap. In addition, the sap freezing points were also clearly lower overall, by *c*. 10 degrees, implying a high purity (and thus a low osmotic force). The concentration of ice‐active particles per milliliter of extracted sap peaks at 10^6^ ml^−1^, or *c*. 1 in 100, when compared to previous nanoparticle tracking measurements. Our results, therefore, suggest that freezing originates on the conduit walls, or possibly interconduit pits, in winter‐hardy branches.

We propose a so‐called ‘Goldilocks model’ to explain the phenomenon: active sites on the conduit surface are ‘deactivated’ by negative pressure, emptying them of water if they are too large. The remaining sites will either activate or not with a probability that is related to the proportion of pores that can accommodate a critical embryo. We find that the model largely replicates the observed freezing points of *Pinus sylvestris* and *Picea abies*, within the range of water potentials up to the point where both trees lose xylem conductance. At more negative water potentials, freezing points appear to level off, suggesting a regime in which multiple freezing events occur independently in disconnected clusters of conduits.

Further work is needed to find whether the model is applicable to the observed freezing behavior in other species and to check whether the fit values of pore sizes are realistic. In particular, it is hoped that extremely high spatial resolution techniques (such as atomic force microscopy) may eventually be used to shed light on the surface roughness/structure of xylem conduit walls in different species. It is also unclear whether other aspects of plant frost tolerance, such as hysteresis, can be captured by such a simplistic model. But we believe that the underlying physical mechanism proposed here is generally applicable: freezing temperature is related to the size of the structures in which ice nucleation occurs. A reduction in that size, or a reduction in the total number of such structures available, results in a decreased freezing point.

## Competing interests

None declared.

## Author Contributions

The project was originally devised by Anna Lintunen. Branch freezing measurements were conducted by AZ, supervised by Anna Lintunen. Sap freezing measurements were conducted by LM and AAP, supervised by Ari Laaksonen. Model development and fitting to experiment were conducted by SI. The paper was primarily drafted by SI, with *Pinus sylvestris* branch freezing section written by Anna Lintunen and Cold stage measurements section by AAP. All authors contributed to finalizing the manuscript.

## Disclaimer

The New Phytologist Foundation remains neutral with regard to jurisdictional claims in maps and in any institutional affiliations.

## Supporting information


**Fig. S1** Scatter plot of sap and branch freezing points against one another.
**Fig. S2** Visualization of the pore size distribution underlying the Goldilocks model during operation.
**Fig. S3** Influence of metastable area and variance on predicted ice nucleation temperatures.
**Notes S1** Derivation of the relationship between water potential, water activity and freezing temperature.
**Table S1** Unaveraged *Pinus sylvestris* branch freezing points.
**Table S2** Averaged *Pinus sylvestris* branch freezing points.
**Table S3** Comparison of branch and sap freezing measurements.
Please note: Wiley is not responsible for the content or functionality of any Supporting Information supplied by the authors. Any queries (other than missing material) should be directed to the *New Phytologist* Central Office.

## Data Availability

Cold stage and branch freezing measurement data are available at the FMI repository and can be downloaded at doi: 10.57707/fmi‐b2share.b0c1e8e4d15b472c8df10984fee85e8a. In addition, a Python script containing the Goldilocks model, as well as files allowing Fig. 4 to be plotted, are provided at doi: 10.57707/fmi‐b2share.58c9429e32004956a6846223f4de31e7.
