# Supplementary material for: Toward understanding apoplastic freezing under negative pressure
Source: New Phytol. 2025 Sep 12;248(3):1245–54. doi: 10.1111/nph.70538 (PMC12489277; doi:10.1111/nph.70538)
Supplement: Supplementary file 1 — Fig. S1 Scatter plot of sap and branch freezing points against one another. Fig. S2 Visualization of the pore size distribution underlying the Goldilocks model during operation. Fig. S3 Influence of metastable area and variance on predicted ice nucleation temperatures. Notes S1 Derivation of the relationship between water potential, water activity and freezing temperature. Table S1 Unaveraged Pinus sylvestris branch freezing points. Table S2 Averaged Pinus sylvestris branch freezing points. Table S3 Comparison of branch and sap freezing measurements. Please note: Wiley is not responsible for the content or functionality of any Supporting Information supplied by the authors. Any queries (other than missing material) should be directed to the New Phytologist Central Office. [file NPH-248-1245-s001.pdf]

New Phytologist

***New Phytologist* Supporting Information**

Article title: Towards understanding apoplastic freezing points under negative pressure
Authors: Ingram, Stephen ; Zanetti, Alessandro; Mustonen, Linnea; Álvarez Piedehierro, Ana; Laaksonen, Ari; Lintunen, Anna
Article acceptance date: 13 August 2025

The following Supporting Information is available for this article:

Notes S1:

The water potential present in a leaf is determined by

$$\Psi_{water} = \frac{k_B T}{v_l} \ln(a_w) \tag{1A}$$

where a_w is the gas phase water activity (humidity) outside the leaf, and v_l is the per molecule volume of water, as in equation 2. From Koop et al. (2000), we have

$$a_w = e^{\frac{\Delta\mu(T,p)}{RT}} \tag{2A}$$

where μ is the temperature and pressure dependent chemical potential, and R is the gas constant. $\Delta\mu$ is the potential difference between the state under consideration and pure water:

$$\Delta\mu(T,p) = \Delta\mu(T,0) + \int v_l\ (p)dp + \int\ v_l^0(p)\ dp \tag{3A}$$

As mentioned in the main text, v_l is only weakly dependent on pressure within the range of p present in tree tissue. As such, it is possible to equate water potential, Ψ_{water}, with chemical potential per unit volume. Substituting equation 2A into 1A then gives:

$$\Psi_{water} v_l N_A = \Delta\mu(T,0) \tag{4A}$$

where N_A is Avogadro's constant. This result is also derivable from the Gibbs-Duhem equation. Equation 2 of Koop et al 2000 provides a parametrization for chemical potential as a function of Temperature. Sap freezing points are therefore predicted to vary according to water potential according to:

$$\Psi_{water}(T) = \frac{1}{v_l N_A}[210368 + 131.438T - 3.32373 \times 10^6 T^{-1} - 41729.1 \ln(T)] \tag{5A}$$

Solving the above for T at individual values of Ψ_{water}, and then scaling it to go through the first experimental data point, produces the green dashed line in Figure 1.

New Phytologist

Koop, T., Luo, B., Tsias, A. & Peter, T. (2000) Water activity as the determinant for homogeneous ice nucleation in aqueous solutions. Nature, 406(6796), 611–614. doi:10.1038/35020537.

URL http://www.nature.com/doifinder/10.1038/35020537

Table S1: Unaveraged *Pinus sylvestris* branch freezing points

Ice Nucleation Temp [°C]	Water Potential [Bar]
-4.549	-2.675
-4.252	-3.025
-4.461	-3.6
-3.73	-5.65
-4.247	-5.525
-5.712	-53.725
-5.35	-42.325
-5.445	-28.8
-4.715	-32.15
-4.574	-37.25
-2.999	-51
-4.128	-30.25
-4.189	-30
-4.902	-63.75
-4.525	-27.375
-2.3	-5.78
-3.375	-5.525
-3.757	-11.157
-3.668	-8.525
-2.4	-9.7
-5.702	-48
-5.731	-54.5
-6.137	-50.25
-7.197	-50.25
-5.991	-65.4
-5.854	-52.75
-6.137	-38.75
-6.108	-43.5
-7.858	-61.25
-7.819	-53
-5.655	-47.25
-4	-22
-4.8	-17.5

-3.9	-15.5
-4.3	-14.7
-4.4	-10.6
-3.6	-10.5
-3.3	-8
-3.2	-7
-2.5	-3.6
-0.5	-1.1

Table S2: Averaged *Pinus sylvestris* branch freezing points (divided into bins of width 6 Bar)

Branch freezing temperature [°C]	Water Potential [Bar]	Freezing temperature error [°C]	Water Potential Error [Bar]
−3.32378	−4.05333	1.26388	1.56179
−3.47500	−9.35459	0.56693	1.42922
−4.33333	−15.90000	0.36818	1.17757
−4.00000	−22.00000	0.00000	0.00000
−4.71967	−28.72500	0.53091	1.07296
−4.42150	−31.20000	0.29350	0.95000
−5.35550	−38.00000	0.78150	0.75000
−5.70375	−45.26875	0.26969	2.40724
−5.95300	−51.82917	1.51972	1.38372
−5.73100	−54.50000	0.00000	0.00000
−6.25033	−63.46667	1.22063	1.70604

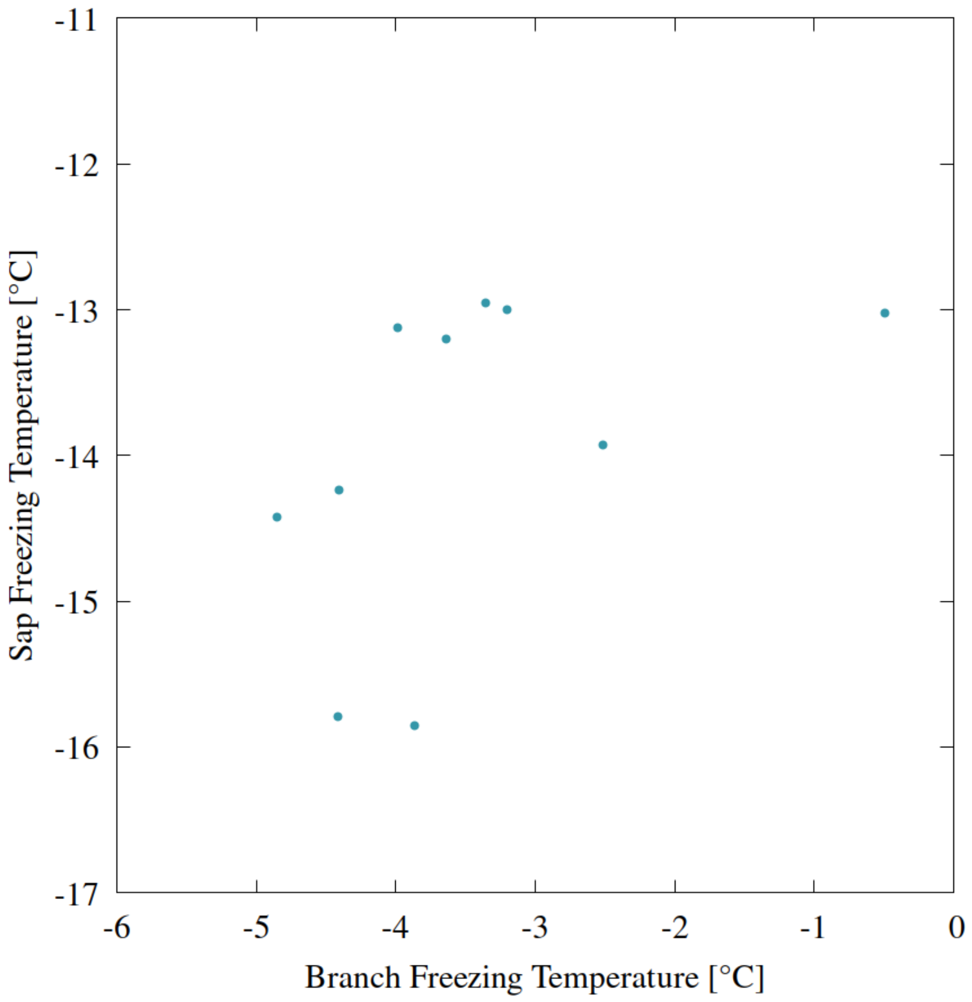

Fig. S1 Scatter plot of sap against branch freezing points. Branch pairs were selected that had water potentials as close to each other as possible. See also supplementary file TableS3.xlsx for full dataset.

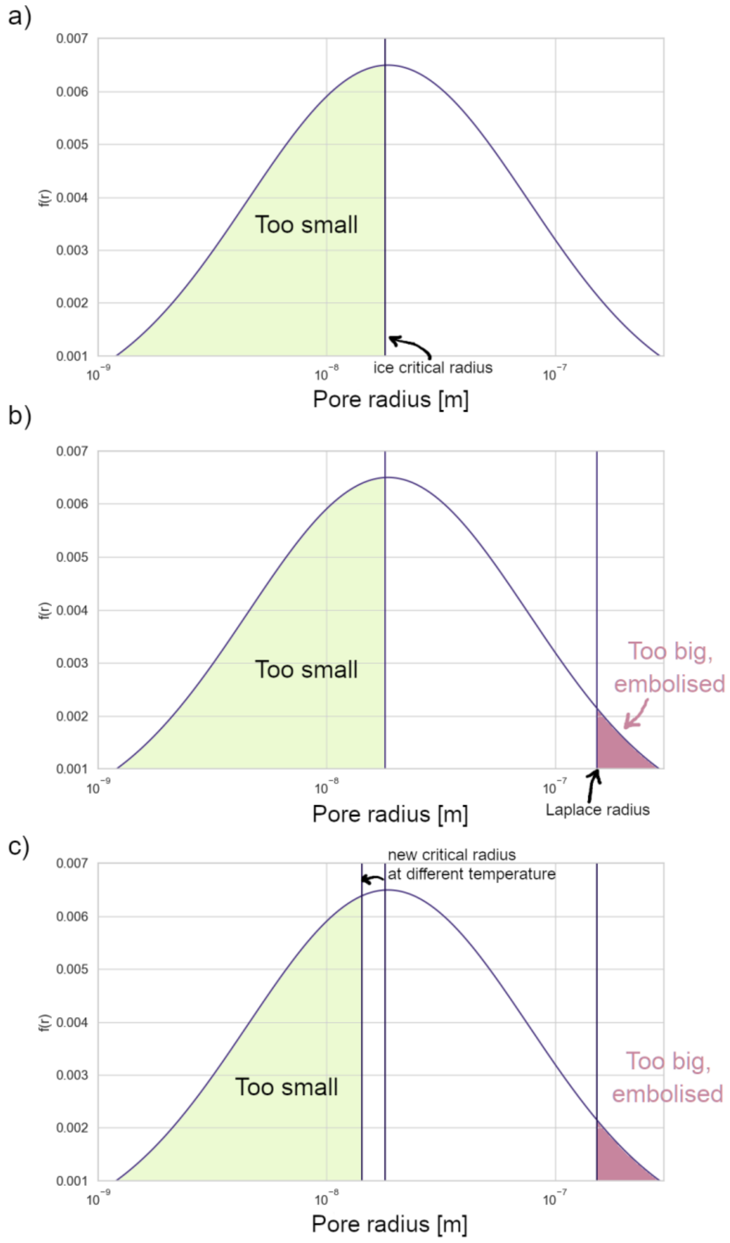

Fig. S2 Visualization of the pore size distribution underlying the Goldilocks model operating in fixed metastability mode. (a) At the onset temperature ($p \approx 0$), the mean pore size is set as the critical ice radius. (b) As pressure becomes more negative, some of the pores are emptied due to being above the Laplace radius. (c) to conserve the total metastable area, the ice critical radius is reduced, and a new temperature calculated.

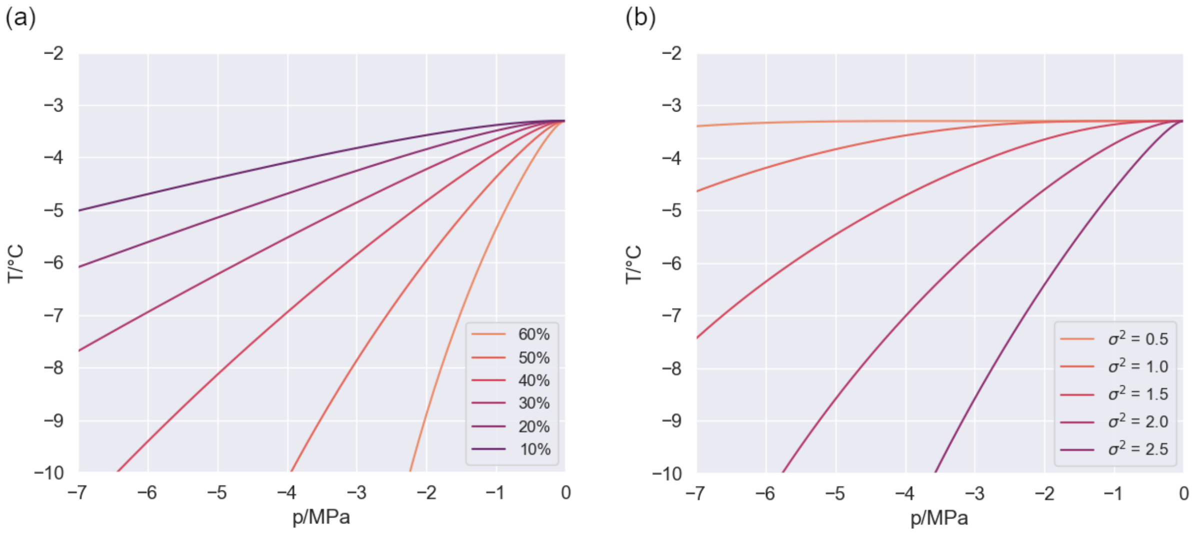

Fig. S3 Influence of (a) fixed metastable area and (b) pore size distribution variance on predicted ice nucleation temperatures by the Goldilocks model. Panel (a) is calculated for a variance of 2.4 logarithmic units and panel (b) for a metastable area of 50%.
